# Constructing Neuronal Network Models in Massively Parallel Environments

**DOI:** 10.3389/fninf.2017.00030

**Published:** 2017-05-16

**Authors:** Tammo Ippen, Jochen M. Eppler, Hans E. Plesser, Markus Diesmann

**Affiliations:** ^1^Institute of Neuroscience and Medicine (INM-6) and Institute for Advanced Simulation (IAS-6) and JARA BRAIN Institute I, Jülich Research CentreJülich, Germany; ^2^Faculty of Science and Technology, Norwegian University of Life SciencesÅs, Norway; ^3^Simulation Laboratory Neuroscience—Bernstein Facility Simulation and Database Technology, Institute for Advanced Simulation, Jülich Research Centre and JARAJülich, Germany; ^4^Department of Biosciences, Centre for Integrative Neuroplasticity, University of OsloOslo, Norway; ^5^Department of Psychiatry, Psychotherapy and Psychosomatics, Medical Faculty, RWTH Aachen UniversityAachen, Germany; ^6^Department of Physics, Faculty 1, RWTH Aachen UniversityAachen, Germany

**Keywords:** multi-threading, multi-core processor, memory allocation, supercomputer, large-scale simulation, parallel computing, spiking neuronal network

## Abstract

Recent advances in the development of data structures to represent spiking neuron network models enable us to exploit the complete memory of petascale computers for a single brain-scale network simulation. In this work, we investigate how well we can exploit the computing power of such supercomputers for the creation of neuronal networks. Using an established benchmark, we divide the runtime of simulation code into the phase of network construction and the phase during which the dynamical state is advanced in time. We find that on multi-core compute nodes network creation scales well with process-parallel code but exhibits a prohibitively large memory consumption. Thread-parallel network creation, in contrast, exhibits speedup only up to a small number of threads but has little overhead in terms of memory. We further observe that the algorithms creating instances of model neurons and their connections scale well for networks of ten thousand neurons, but do not show the same speedup for networks of millions of neurons. Our work uncovers that the lack of scaling of thread-parallel network creation is due to inadequate memory allocation strategies and demonstrates that thread-optimized memory allocators recover excellent scaling. An analysis of the loop order used for network construction reveals that more complex tests on the locality of operations significantly improve scaling and reduce runtime by allowing construction algorithms to step through large networks more efficiently than in existing code. The combination of these techniques increases performance by an order of magnitude and harnesses the increasingly parallel compute power of the compute nodes in high-performance clusters and supercomputers.

## 1. Introduction

Simulation has become an essential part of the scientific method. In neuroscience, it is employed to investigate the relationship between anatomical and physiological data, to explore dynamical systems not accessible by analytical methods, and to validate approximations made in theoretical derivations. This was made possible by progress in computer hardware as well as in simulation technology for models ranging from the molecular dynamics of ion channels via detailed compartmental models of individual nerve cells (neurons) to brain-scale networks of simple neuron models and field models. Today, simulation codes exist for all of these levels, but the degree of usage by the community varies (Carnevale and Hines, 2006; de Kamps et al., 2008; Helias et al., 2012; Hepburn et al., 2012; Ritter et al., 2013).

Nerve cells interact primarily through stereotyped point events called action potentials or spikes, which are transmitted unidirectionally with delay from sending to receiving cell through contacts known as chemical synapses. After the sending (presynaptic) neuron emits a spike, the receiving (postsynaptic) neuron experiences an excursion of the electric potential difference between the inside and the outside of the cell, the postsynaptic potential (PSP). Typically on the order of ten to one hundred PSPs need to arrive within a few milliseconds in order to elicit an action potential in the postsynaptic neuron (see Abeles, 1991; Sterratt et al., 2011, for textbooks).

The essential challenge for a simulation code aiming to implement brain models based on simplified neuron and synapse models is the number of network elements that need to be represented in the computer. In the mammalian cortex, a neuron receives some 10, 000 local inputs. Since two neurons form a connection with a probability of 0.1, the smallest network in which both constraints are simultaneously fulfilled already has 100, 000 neurons. The corresponding volume of roughly one cubic millimeter of tissue can be considered as an elementary unit of cortex. However, the local connections constitute only about half of the input to a neuron, while the other half originates from more distant locations (see Potjans and Diesmann, 2014, and references therein). A substantial fraction of these long-range connections directly links neurons from different areas in the brain. The human brain is divided into some two hundred areas per hemisphere, but an individual area is only connected to a fraction of them (Glasser et al., 2016). The brain thus forms recurrent networks at multiple levels of organization, and due to this intricate coupling between the local and the global level neuroscientists need to study brain-scale networks in order to arrive at self-consistent descriptions of brain activity.

In the past decade, research on simulation technology for spiking neuronal networks focused on data structures capable of representing networks of increasing size. Morrison et al. (2005) presented the first code capable of full-scale simulation of local cortical networks, representing the 100, 000 neurons with their one billion synapses, using distributed computing to aggregate the memory from some ten compute nodes (see also Migliore et al., 2006, for work carried out at about the same time). Whether downscaling or dilution of neuronal networks preserves the dynamical state of a neuronal network model has been a matter of debate. Recently van Albada et al. (2015) found that the first-order statistics (e.g., spike rates) can excellently be maintained, but distortions occur already for second-order statistics (e.g., correlations). This observation is relevant not only because correlations of spiking activity are an important measure for the experimentalist and impact spike-timing dependent plasticity (STDP), but also because correlations in neuronal activity drive fluctuations on the population level and thus determine meso- and macroscopic measures such as the local field potential (LFP) and the EEG (Lindén et al., 2011; Tetzlaff et al., 2012).

In light of the limited explanatory power of downscaled network models, the technology of Morrison et al. (2005) represents a breakthrough, because at the scale of 100, 000 neurons each neuron is supplied with the number of synapses found in nature. Larger networks are necessarily less densely connected, and therefore from this threshold on memory consumption grows linearly with network size, instead of quadratically as is typical for down-scaled networks (see Lansner and Diesmann, 2012, for details). Morrison and colleagues already point out that the time required to construct a neuronal network model in the main memory of the computer may take up a considerable fraction of the total simulation time and therefore one should make use of all the compute power available. They show furthermore that network construction is ideally parallelizable for a class of network structures. Their technology enables new findings on the dynamics and function of local cortical networks to the present day (Potjans and Diesmann, 2014). Due to the progress in computer hardware, networks of 10, 000 neurons are today comfortably studied on a laptop and networks of 100, 000 neurons just require one node of an HPC cluster.

Since 2005 (Morrison et al., 2005), improvements in neuron (Kunkel et al., 2012) and connectivity (Kunkel et al., 2014) representation in simulations of networks of spiking neurons have expanded the range of brain models that can be simulated on available computing hardware. The focus of those studies is to minimize memory requirements without sacrificing performance in the propagation of the network state for a given span of biological time. Using supercomputers, networks with more than one billion neurons and the corresponding number of synapses can now be simulated. This already exceeds the number of neurons in the brain of a mouse (100 million), but is still two orders of magnitude away from the number of neurons of the human brain (100 billion). These advances enable the construction of multi-area models addressing the lack of self-consistency in local cortical network models mentioned above and making the link to meso- and macroscopic observables. First neuroscientific results are emerging (Schmidt et al., 2016).

With the problem of network representation being solved for the range of systems from laptops to petascale supercomputers, increasing the speed of simulations becomes an urgent issue. Simulation times for brain-scale networks are orders of magnitude slower than real time, ruling out the investigation of plasticity and learning which span minutes and hours of biological time. As a first step toward faster simulation, we focus on the time required to create instances of neuronal network models in the main memory of modern computing hardware.

We distinguish between two different simulation use cases that we address in this paper, both of which depend on fast network instantiation. One use case is the rapid exploration of the parameter space defining network model properties, including systematic parameter optimization (Martínez-Cañada et al., in press). This is useful to identify parameter ranges for which a network model shows stable behavior, and typically combines network instantiation with a short simulation run covering on the order of a second of biological time. This use case requires that instantiation is not significantly slower than simulation. The other use case are models at the scale of entire brains, filling petascale supercomputers with on the order of 100, 000 CPU cores. Network construction time on these systems is presently in the range of 15 min (Kunkel et al., 2014). Compute time on such systems is a limited commodity and should not be wasted on sub-optimal network instantiation. Furthermore, entire compute nodes or even racks are often allocated for single jobs, implying that usage is only efficient if a simulation employs all processor hardware available in these units.

As we focus on network instantiation in this work, especially on connection instantiation, we limit ourselves to simplified neuron models, describing the dynamics of a neuron by a small number of—often linear—ordinary differential equations combined with a threshold and reset mechanism representing the generation of action potentials. Our results apply also to more complex neuron models, including multicompartment models, provided that significantly less memory is required to represent neurons than synapses, and that the number of synapses is much larger than the number of neurons. We further confine our investigation to the creation of connections representing chemical synapses. While neurons interact also by other biophysical mechanisms, some of which are supported by current simulation technology (see Hahne et al., 2015 for electrical synapses (gap junctions) and Potjans et al., 2010 for neuromodulatory control of synaptic plasticity), these do not pose any new challenges for network instantiation from a simulation technology perspective.

In practice, neuroscientists formulate their simulation setups using a high-level programming language designed for expressiveness in the problem domain. Research on suitable languages is ongoing. In common use are variants embedded into the Python language (Davison et al., 2008; Eppler et al., 2008) which has become a de facto standard in computational neuroscience (Muller et al., 2015). Network models commonly use combinations of deterministic and probabilistic rules to specify the connectivity among subpopulations of neurons (Crook et al., 2012). The script specifying a simulation essentially consists of a sequence of collective Create() commands for the instantiation of populations of different cell types and collective Connect() commands establishing and parameterizing the corresponding synapses. The specifications of the use cases in the present work follow this approach to perform all analyses under realistic conditions.

Modern computing hardware beyond the desktop computer typically consists of a number of compute nodes connected by a fast interconnect such as Infiniband. Each compute node contains a number of CPUs, which in turn contain a number of cores that execute instructions. Since all cores within a single compute node share a common main memory and are managed by a single instance of the operating system, it is possible to parallelize simulations within a compute node using threads. A particular specification of a programming model for multi-threading in widespread use is OpenMP (OpenMP Architecture Review Board, 2008). Parallelization across multiple compute nodes, on the other hand, requires communication over a physical network. In common use is the message passing interface MPI (Message Passing Interface Forum, 2009).

As MPI-based parallelization commonly incurs a memory and communication overhead compared to thread-based parallelization, a combination of both technologies is desirable. Initial work concentrating on the phase were the state of the network is advanced in time was already carried out a decade ago (Plesser et al., 2007). Here, each MPI process is split into a number of threads and each such thread is called a virtual process (VP). The present work builds upon these early explorations.

We consider first the time required to simulate a neuronal network model of a size typically used in computational neuroscience today. The computer is a single multi-core system commonly used in theoretical laboratories. We call this network model small, because it represents only 25% of the neurons within the reach of the local connectivity in the mammalian cortex and only 6.25% of the one billion synapses in a cubic millimeter of cortex.

**Figure 1:**
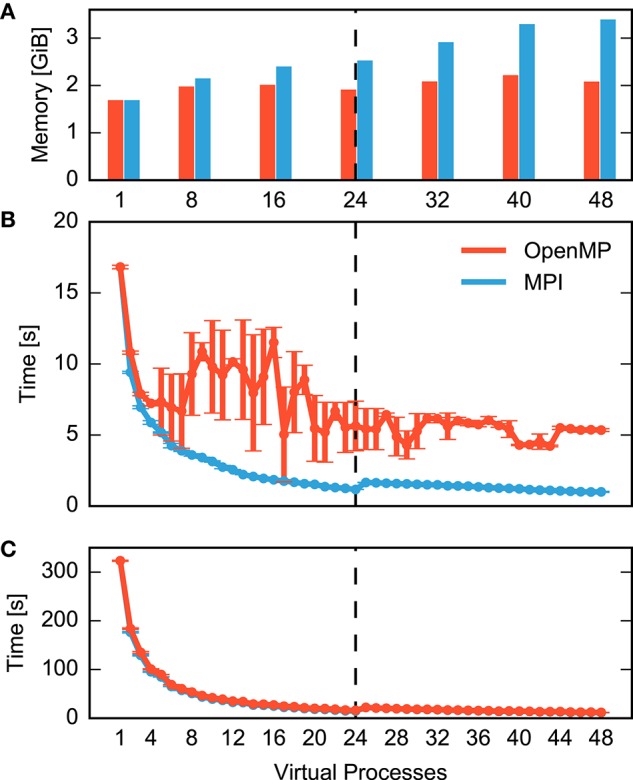
**Performance of a small neuronal network model on a single shared-memory compute node**. A balanced random network model (Brunel, 2000) representing 25,000 neurons and 62.5 million synapses is simulated for one second of biological time (small benchmark). The compute node houses two CPUs with 12 cores each and up to two hardware threads per core. Table 1 summarizes the configuration. For detailed system specifications see Sections 2.2.1, and 2.2.2 for model specifications. **(A)** Memory consumption and **(B)** runtime of network construction as a function of the degree of parallelization. Red indicates parallelization using OpenMP threads and blue using MPI processes. Virtual processes first bind to cores on one CPU (up to 12 VPs), then on the second CPU (up to 24 VPs), and finally to the second hardware thread on each core (up to 48 VPs). The data are averages over five simulations with identical seeds of random number generators. Error bars in **(B)** show one standard deviation of measurements. **(C)** Runtime of the simulation of network dynamics, excluding the network construction phase shown in **(B)**. Same notation as in **(B)**. The dashed vertical line indicates the total number of physical cores of the compute node.

Figure 1 compares MPI- and OpenMP-based parallelization and separates the total time for a simulation run into the time required to construct the network (Figure 1B) and the time it takes to simulate the network, i.e., to advance the dynamical state of the network over the desired span of biological time (Figure 1C). Simulation time declines with increasing number of processes for both MPI (blue) and OpenMP (red) until the simulation exhausts the number of computational cores (24). In spite of hardware support for two parallel processes per core (hyperthreading), simulation times increase at first when using more than 24 processes. Even with 48 processes, simulation times are only about 25% shorter than with 24 processes. Still, simulation time is reduced from over five minutes for a single process to slightly more than ten seconds for 48 processes.

Network construction (Figure 1B) shows very different scaling for MPI and OpenMP. When using OpenMP, construction times decline markedly only for up to four threads, followed by a complex non-monotonic course and saturate at a network construction time about five times as long as with MPI. Parallel processes do not communicate during network construction and therefore there is a priori no reason why the two parallelization schemes should exhibit different runtime performance.

It is instructive to inspect memory consumption shown as a bar diagram in Figure 1A. While memory consumption is independent of parallelization when using OpenMP, it increases when using MPI, exceeding OpenMP by more than 60% for high degrees of parallelization. The scenario may just be indicative of the common runtime vs. memory consumption dilemma. Thus, we need to find out whether the more compact representation enabled by the shared memory access of OpenMP incurs runtime costs due to the need to coordinate access to joint data structures.

**Figure 2:**
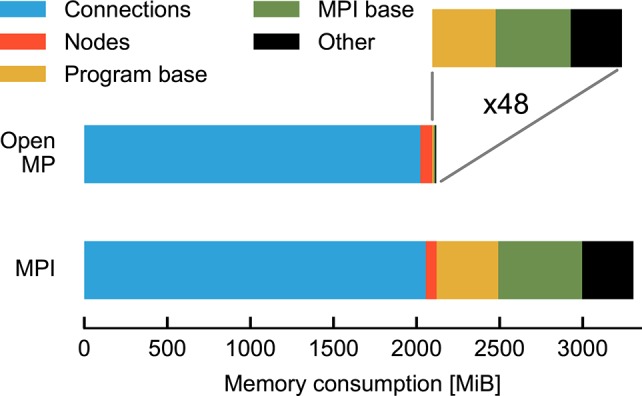
**Map of memory consumption at the end of the network construction described in Figure 1**. For parallelization with OpenMP (top) 48 threads are used and, correspondingly, for MPI (bottom) 48 processes (rightmost data points in Figure 1). The absolute contributions to the total memory consumption are distinguished by color from left to right. In the case of OpenMP these are: synapses (blue, 2 *GiB*), neurons (red, 72 *MiB*), overhead at start of program (yellow, 6.4 *MiB*), initialization of MPI with MPI_Init() (green, 9.4 *MiB*), and other NEST data structures (black, 7.9 *MiB*). MPI_Init() is not required for OpenMP parallelization and shown for comparison with MPI parallelization only. The enlargement (indicated by gray lines) shows the latter three contributions (total 23.7 *MiB*) multiplied by 48 (1138 *MiB*). In the case of the MPI simulation the data are sums over all processes, the last three components occupying a total of 1184 *MiB*. Memory consumption is measured by the resident set size (see Section 2.5).

Figure 2 compares the breakdown of memory consumption for the two parallelization schemes at the highest degree of parallelization studied in Figure 1. The major part of memory is occupied by synapses and neurons and is of equal absolute size in both schemes. The discrepancy is explained by the overhead for running multiple processes and the data structures of MPI. While in the OpenMP scheme there is only one process, the overhead is multiplied by a factor of 48 for MPI. As the analysis shows that OpenMP does in fact not reduce the memory required for the representation of the network, the runtime vs. memory dilemma does not explain the inferior runtime of OpenMP.

This is the puzzle we need to resolve. Why does network construction in the OpenMP scheme scale so much worse while it does not use a more compact representation of the network in memory and exhibits no disadvantage in the propagation of the dynamical state? This question is relevant because on future computer systems with hundreds of computational cores but limited amounts of memory per core, we cannot afford to spend the major part of memory on the overhead. A lightweight parallelization scheme is required. At the same time, however, without a corresponding scaling of construction time, systems with many cores will not be of use either. As we can demonstrate excellent scaling using the same representation of the network on identical computer hardware, there seems to be no room for a fundamental obstacle in simultaneously achieving efficient usage of memory and scaling. In this study we explain the observed poor scaling for OpenMP and show how to eliminate this bottleneck.

The remainder of this paper is organized as follows: In the methods section we first specify the simulation software our quantitative data refer to. Next, we describe in detail the neuronal network models and the computer systems used throughout the present work. In order to validate the generality of our conclusions we extend the benchmark scenario indicated in Figure 1 to a large neuronal network model executed on a supercomputer with thousands of compute nodes, where each compute node harbors only a minor fraction of the neurons. Subsequently, we introduce the state-of-the-art high-level data structures representing a network instance in main memory. When these data structures are created, the simulation engine needs to acquire suitable pieces of memory from the operating system. Section 2.4 explains commonly used allocation strategies and how an application can select between them. Finally, the methods section describes our protocol of obtaining quantitative data and the tools for assessing runtime and memory consumption.

In the results section we present an analysis of contributions of different components of the software to the total time required for network construction. For small networks, the creation of connections between neurons dominates, but in the large network setting other components consume the major fraction of runtime. We then show that the standard method of memory allocation serializes the creation of connections when using OpenMP and explain the strategy of advanced allocation algorithms to overcome this without loss of performance for the simulation of the network dynamics. Subsequently we turn to large networks distributed over thousands of compute nodes and exhibit the loops over target neurons that only rarely find local targets as the bottleneck. The reorganization of critical loops eliminates this bottleneck, and combined with thread-aware allocators ensures excellent scaling also for the large benchmark.

In Section 4 we discuss our findings in light of the upcoming massively-parallel compute nodes and exascale computers. The technology described in the present article is available in the open-source simulation software NEST version 2.12 (Kunkel et al., 2017). The conceptual and algorithmic work described here is a module in our long-term collaborative project to provide the technology for neural systems simulations (Gewaltig and Diesmann, 2007).

## 2. Materials and methods

### 2.1. Simulation code

NEST, The Neural Simulation Tool (Gewaltig and Diesmann, 2007; Plesser et al., 2015) is a neuronal network simulation code optimized for large networks of spiking model neurons with relatively simple internal dynamics. In this framework, neuronal networks are directed graphs with nodes representing neurons and stimulation and recording devices, while edges represent synapses between neurons and connections between neurons and devices. NEST provides a range of model neurons and synaptic dynamics, including spike-timing dependent plasticity (STDP). Researchers create network models and specify simulations using high-level commands of a built-in interpreter (SLI), a Python interface (Eppler et al., 2008; Zaytsev and Morrison, 2014), or the PyNN network simulator interface (Davison et al., 2008).

Internally, the simulation kernel, including all neuron and synapse models, is implemented in C++. Neurons and synapses are represented as instances of respective model classes, with an emphasis on efficient neuron lookup and storage of graph connectivity. To facilitate the simulation of brain-scale networks represented by graphs of $O\left(10^{9}\right)$ nodes (neurons) and $O\left(10^{13}\right)$ edges (synapses), the code distributes the memory required for network connectivity across many compute nodes (Kunkel et al., 2014). For optimal exploitation of computer capabilities, a hybrid parallelization scheme (Plesser et al., 2007) combines MPI (Message Passing Interface Forum, 2009) for process-based with OpenMP (OpenMP Architecture Review Board, 2008) for thread-based parallelism. The concept of virtual processes considers a simulation to be distributed over *VP* = *M* × *T* entities, where *M* is the number of MPI processes and *T* the number of OpenMP threads per MPI process, and guarantees identical results for fixed *VP*, independent of the partitioning between processes and threads. This insulates the neuroscientist from parallelization details.

Global identifiers (GID) linearly enumerate network nodes and distribute them among the virtual processes in a round-robin fashion. This allows for a compact representation of nodes across parallel processes, since each process only needs to know details about local nodes and implements a simple load balancing scheme (Morrison et al., 2005). For non-local nodes, only information on the model class is retained (Kunkel et al., 2012). Edges are represented solely on the virtual process responsible for updating the edge target (Morrison et al., 2005) using a specialized adaptive data structure minimizing memory overhead even in the case of scaling to more than 500, 000 virtual processes (Kunkel et al., 2014); see Section 2.3 for details.

The benchmark data reported here are created using revision 38a9608 of NEST, available publicly on GitHub (https://git.io/v1sHv), except for data on the alternative loop order (Listing 3, Section 3.2) first implemented in revision 5e5cdd7 (https://git.io/v1sHI, **Figures 6**, **7**).

### 2.2. Benchmark scenario

#### 2.2.1. Computing hardware

Computational neuroscientists use simulations across a range of computers, from low-end laptops for education and small-scale simulations to brain-scale simulations on the largest supercomputers available today (Kunkel et al., 2014). In the present study, we explore the performance on two computer architectures typical for demanding simulations: a compute node of a modern high-performance cluster as used for parameter scans on networks with biologically realistic connectivity ($O\left(10^{5}\right)$ neurons, Potjans and Diesmann, 2014), and a supercomputer as required for spiking neuron simulations of brain-scale networks (Schmidt et al., 2016). The details of the computer systems are given in Table 1.

**Table 1:** **Parameters and properties of the small and the large benchmark**.

	**Small**	**Large**
**COMPUTERS**		
System	x86 HPC Cluster	IBM BlueGene/Q
Partition	Compute node	Full supercomputer
Processor	Intel Xeon E5-2680v3	IBM PowerPC A2
Clock (GHZ)	2.5	1.6
CPU/node	2	1
Cores/CPU	12	16
Hardware threads/core	2	4
Hardware threads/node	48	64
Memory/node	128 *GiB* DDR4	16 *GiB* DDR3
Memory/hardware thread (GiB)	2.7	0.25
OS	CentOS Linux 7.1.1503	CNK
Compiler	GCC v4.8.3	GCC v4.4.7
MPI	OpenMPI v1.10.0	MPICH v1.5
OpenMP API version	OpenMP 3.1	OpenMP 3.1
**NETWORK PARAMETERS**		
*N*	25, 000	2 × 10^8^
*K*	2, 500	11, 250
ϵ	0.8	0.8
**BENCHMARK PROPERTIES**		
*NK*	62.5 × 10^6^	2.25 × 10^12^
*NVP*	521	109
*KVP*	1, 302, 083	1, 226, 152
*N∅c*	0	198, 777, 598
*N*1*c*	0	1, 218, 658
*N*>1*c*	25, 000	3, 743

*The top part specifies the computers used for the two benchmarks and the center part characterizes the respective neuronal networks. The high-performance cluster and the supercomputer architecture execute the same network model with adapted total number of neurons N, in-degree K, and share of excitatory neurons ϵ. The bottom part summarizes properties of the benchmarks derived from combinations of computer and network parameters: total number of synapses NK, number of local neurons per virtual process N_vp_, number of local synapses per VP KVP, expected number of source neurons without targets, $N_{c}^{\emptyset }$, with exactly one target, $N_{c}^{\text{1}}$, and with more than one target, $N_{c}^{>\text{1}}$, on a given VP. For derivation of the quantities, see Kunkel et al. (2014) (Section 2.4) Calculations assume use of the maximum number of hardware threads per compute node (48, 64) and, for the large benchmark, use of 28, 672 compute nodes*.

#### 2.2.2. Neuronal network model

All benchmarks reported in this study are performed by constructing and simulating a variant of a widely-used balanced random network model (Brunel, 2000), which has also been used in previous benchmarking studies (Morrison et al., 2007; Helias et al., 2012; Kunkel et al., 2012, 2014), and is available in NEST 2.12.0 as hpc_benchmark.sli (Kunkel et al., 2017).

The network consists of ϵ = 80% excitatory and 1 − ϵ = 20% inhibitory neurons driven by external random spike trains. Each neuron receives input from *n*_E_ excitatory and *n*_I_ inhibitory neurons, which are chosen at random from the respective cell types for a total number of *n* = *n*_E_ + *n*_I_ inputs, also called the in-degree. The total number of neurons *N* and the in-degree *n* are scaled to adjust the network size to the compute node and supercomputer hardware. During simulation, this network exhibits asynchronous irregular spiking activity at a stationary average firing rate. Connections between excitatory neurons exhibit spike-timing-dependent plasticity (Morrison et al., 2007), while all other connections are static. The Supplementary Material includes the simulation scripts generating the raw data of the present study.

Table 1 summarizes the parameters of the network model scaled to compute node and supercomputer sizes. The table also shows the expected number of neurons and synapses on each virtual process, as well as details of the connectivity data structures discussed in detail in Section 2.3.

### 2.3. Representation and instantiation of network structure

#### 2.3.1. Representation and iteration

Neuroscientists specify network structure by deterministic or probabilistic rules. We discuss here how a simulation code decides which nodes to connect, while Section 2.3.2 describes the layout of the adjacency table representing the edges of the instantiated network in the main memory of the computer system.

The basic principle of network instantiation is that the virtual process responsible for updating the state of a particular neuron is also responsible for creating all incoming connections of this (postsynaptic) target neuron and for representing them in its share of the main memory of the computer; see Morrison et al. (2005) for details and justification.

The instantiation of a network on the basis of connection rules conceptually consists of the following steps:

1. Specify ordered sets of potential source and target neurons.
2. Specify a synapse model, possibly with parameters.
3. Specify a connection rule, possibly with parameters.
4. Perform preparations, which may entail communication between MPI processes.
5. Iterate over source or target sets, or both, in a suitable fashion and select source-target pairs to connect.
6. Create, parameterize, and store connection objects for all selected pairs.

In order to illustrate how the iteration is organized in parallel network construction, we limit our investigation to the fixed in-degree rule because of its prominent role in computational neuroscience in the last two decades (Amit and Brunel, 1997; Brunel, 2000) and because it parallelizes perfectly in the simulation engine architecture introduced in Section 2.1. The fixed in-degree rule thus exposes the effect of loops over network elements and of memory allocation costs on performance without distraction by communication between compute nodes during network creation or other overheads.

Listing 1 shows the details of the fixed in-degree implementation. It iterates over all neurons in the target population in parallel, with each VP skipping those target neurons that are not managed by the VP. *K* source nodes are then chosen at random and each is connected to the target neuron by a call to the connect(source, target) function which registers the actual connection with the simulator kernel as described in Section 2.3.2. Here, we ignore details, such as parameterization of the connection (weight, delay, etc.), compatibility checks between source and target, and checks preventing multiple connections for a given source-target pair (multapses) or self-connections (autapses), since they do not relate to the focus of the present work.

**Listing 1:**
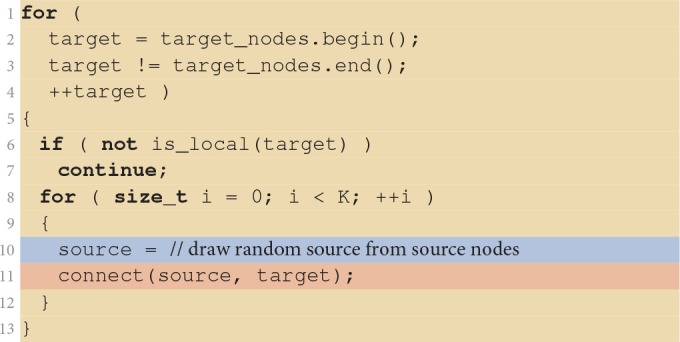
**Implementation of the fixed in-degree rule**. The algorithm is executed in parallel by all virtual processes. If a target is not local to the virtual process (line 6) it is skipped. Otherwise, the virtual process randomly selects (line 10, blue), one by one, *K* of the source nodes (lines 8–12) and connects them to the target (line 11, red). Iteration code is shown on yellow background.

#### 2.3.2. Instantiation in main memory

The simulation engine stores connections when a connection algorithm calls connect(source, target); for details of the underlying data structures and algorithms see Kunkel et al. (2014). Briefly, each virtual process stores all connections with targets local to the process in a hierarchical data structure, mapping source neurons to connections. We need to consider four cases:

1. A source neuron has no targets on a VP. This is marked by a single zero bit in a sparse table (Silverstein, 2005).
2. A source neuron has fewer than *K*_cutoff_ = 3 targets on a VP and all use the same synapse model. The connections are stored in a homogeneous fixed-size connector, implemented as a plain C-style array with one element per target.
3. A source neuron has *K*_cutoff_ or more targets on a VP connected using the same synapse model. The connections are stored in a homogeneous variable-size connector, implemented as a C++ STL vector.
4. A source neuron has targets connected by different synapse models. Connections are represented by a heterogeneous connector, a C++ STL vector containing one homogeneous connector per synapse model.

Table 1 displays the characteristic quantities of the benchmark networks for the maximum number of VPs per compute node and shows that cases 1 and 2 dominate for parallel simulations of large networks.

Listing 2 sketches how a new connection is registered in a homogeneous connector. The current connector of the source neuron is obtained from the sparse table of the VP. If the sparse table does not contain a connector, the algorithm creates a connector of size *K* = 1 and stores the particular connection. If the present size *K*_old_ of the connector is below *K*_cutoff_, the procedure creates a new connector of size *K*_old_ + 1, copies all existing connections, stores the new connection, and finally deletes the old connector. The template argument of the Connector class (see caption of Listing 2) selects the implementation of the connector. If the new size *K*_old_ + 1 equals *K*_cutoff_, a connector with a dynamic number of connections is created instead of the size-specific connectors used for lower numbers of connections. Consequently, a connector with *K*_cutoff_ or more elements is dynamic in size and the algorithm simply appends the new connection. Finally, the procedure registers the new connector for the source in the sparsetable. Dedicated templatized Connector types for very short target lists do not increase the complexity of the first steps of connector growth, because the vector push_back() operation on line 22 of Listing 2 encapsulates an operation of similar complexity as the explicit Connector replacement on lines 14–17.

**Listing 2:**
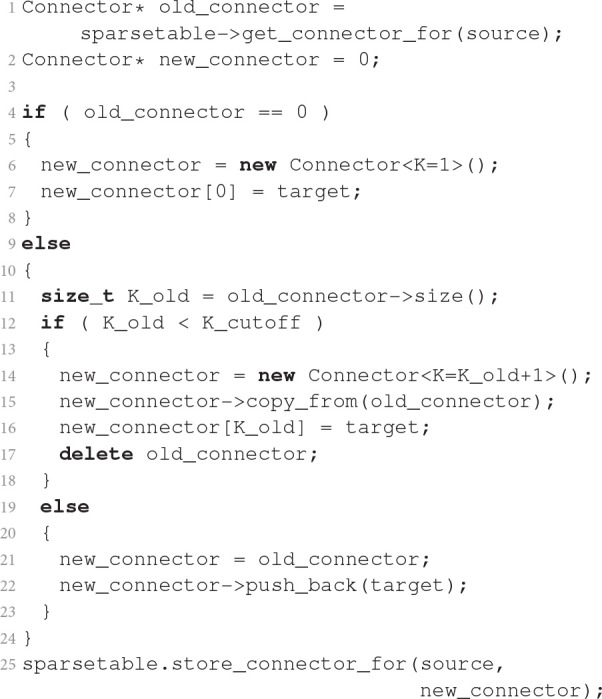
**Dynamic selection of container class in dependence on number of connections**. The simulation engine executes this procedure every time it stores a single connection from source to target. The listing states the algorithm in pseudocode close to the C++ implementation. The angle brackets enclose integer template arguments. These enable access to different implementations of the class through specialization. For simplicity the code assumes that the Connector is homogeneous before and after the new connection is added. Furthermore, the connector stores only target, the downstream GID, neglecting potential further parameters of the connection such as weight and delay.

Next we estimate the number of memory allocations and deallocations (frees) required to build a network. We consider the large benchmark first. Each neuron has a fixed fan-in of *K* = 11, 250 (see Table 1) and by symmetry on average *K* = 11, 250 outgoing connections. Each connection is represented only on the virtual process on which its target neuron resides. For *M* = 28, 672 parallel processes a source neuron has on average 0.006 (*T* = 64 threads per process) to 0.4 (*T* = 1) connections per virtual process, so that on any given virtual process almost all source neurons either have no connection at all or only a single connection. Let *N*1*c* be the number of source neurons on a virtual process with one connection. Storing this connection requires two steps: creating a homogeneous one-element homogeneous connector (one allocation) and registering it in the sparsetable, which in turn requires one free and one allocation. The total number of memory operations on each virtual process is thus approximately

(1) $$n_{\text{alloc}}\approx 2N_{\text{c}}^{1}$$

(2) $$n_{\text{free}}\approx N_{\text{c}}^{1}.$$

For the small benchmark, we need to consider that excitatory neurons connect to excitatory and inhibitory targets with different synapse types, and thus require a heterogenous connector containing one homogeneous connector for each synapse type. Since ϵ*N* neurons are excitatory, each neuron has outgoing connections to on average *K*_E_ = ϵ*K* excitatory and *K*_I_ = (1 − ϵ)*K* inhibitory neurons, which are distributed across all *VP* virtual processes. The average connector size is then for excitatory-excitatory connections

(3) $$k_{\text{EE}}=\frac{\varepsilon K}{\text{VP}},$$

for excitatory-inhibitory connections

(4) $$k_{\text{EI}}=\frac{\left(1-\varepsilon \right)K}{\text{VP}},$$

and for all connections from inhibitory neurons

(5) $$k_{\text{I}}=\frac{K}{\text{VP}}$$

as these use only a single synapse type and thus store all outgoing connections in a single homogenous connector. We find the shortest connectors for excitatory-inhibitory connections at the maximum number of 48 VPs, in which case *k*_EI_ ≈ 10.4 ≫ *K*_cutoff_ = 3. This means that in the small benchmark all connectors are eventually stored using dynamically sized containers.

Registration of the first connection for any source neuron again requires one allocation and one free for sparsetable registration and one allocation for the initial one-element homogenous connector. This is replaced by a two-element connector for the second connection (one allocation, one free) and a dynamically-sized connector for the third connection (one allocation, one free). Beyond this point, details of memory management depend on the allocation strategy of the underlying dynamic container. We assume that it doubles its capacity when necessary, so that push_back() has amortized constant complexity (Goodrich et al., 2011, Ch. 6.1.3). For an eventual connector size *k* this requires ⌈log_2_*k*/*K*_cutoff_⌉ doublings with one allocation and one free each. For excitatory source neurons, registration of the first synapse of the second type requires replacing the initial homogenous connector by a heterogenous one at similar cost as registration in the sparsetable so that we can use the same operation count estimate for connectors for both synapse types. Creating the connectors for *S* source neurons with an average connector size *k* therefore requires

(6) $$n_{\text{alloc}}\approx 2S+2S+S\left\lceil \log_{2}\frac{k}{K_{\text{cutoff}}}\right\rceil =\left(4+\left\lceil \log_{2}\frac{k}{3}\right\rceil \right)S$$

(7) $$n_{\text{free}}\approx S+2S+S\left\lceil \log_{2}\frac{k}{K_{\text{cutoff}}}\right\rceil =\left(3+\left\lceil \log_{2}\frac{k}{3}\right\rceil \right)S$$

allocation and free operations. These values are approximations, because the exact number of reallocations required depends on the precise length of the target lists which varies for random networks.

### 2.4. Memory allocators

When a program written in C (or a language derived from it) requires additional memory during runtime, it requests that memory from the operating system by calling the function malloc() or one of its variants (Kernighan and Ritchie, 1988). After the memory is no longer needed, the program returns the allocated memory with free(). When multiple threads simultaneously read from or write to the same memory location, race conditions may occur leading to unpredictable and possibly incorrect behavior. For the same reason, simultaneous attempts to allocate memory lead to overlapping memory regions being handed out to different threads or an otherwise corrupted state of malloc (**Figure 4A**). The default code implementing malloc and free in glibc (Free Software Foundation, 2016) on Linux is ptmalloc (Gloger, 2006), based on the work of Doug Lea (Lea, 2000; Kerrisk, 2010). The algorithm of ptmalloc solves these concurrency issues by guards that limit access to the allocator at the level of the interface functions to one thread at a time, as illustrated in **Figure 4B**; while one thread is executing malloc() any other thread calling the function needs to wait until the first thread is done.

Alternative memory allocation frameworks that focus on multi-threaded performance improve on ptmalloc: A straightforward but rather inflexible design maintains a private memory region for each thread. These regions do not need to be guarded, as only the designated thread accesses a region. Most memory allocations and frees are performed on the private regions. In a second, more flexible design, all threads share a memory pool that is accessed when no suitable piece of memory is available in the private region of a thread. In this case, chunks of memory larger than the typical size requested by a single call to malloc() are exchanged between the memory pool and the private region. This interaction happens seldomly but needs to be guarded, as other threads also have access to the pool. Lastly, an advanced design improves the behavior for small memory requests, i.e., for objects comparable in size to the administrative information required for each allocation (Berger et al., 2000). Here, the allocator not only satisfies the immediate request, but allocates in advance several objects of the same size in a contiguous array. The size of the individual object is stored only once, saving one to two words per object, i.e., 4–16 byte depending on the computer architecture and implementation. When the application allocates and deletes objects of heterogeneous sizes, this strategy also reduces the fragmentation of memory. The downside of this approach is that an application can have an increased memory footprint if the memory access pattern diverges from the described (and expected) behavior.

We focus here on a set of widely used allocators that have a proven track record in the field: tcmalloc, part of Google's gperftools version 2.5 (Ghemawat and Melange, 2007); jemalloc version 4.2.1, the default allocator for FreeBSD's libc since 2005 (Evans, 2006) and used by, e.g., Facebook (Evans, 2011); and the allocator included with Intel's Threading Building Blocks (TBB) library version tbb44_20160526oss (Hudson et al., 2006; Kukanov and Voss, 2007). These allocators can be used with existing simulator code by dynamic preloading of or static linking against the allocator library. We do not consider allocators that are in a research or prototype stage, such as streamflow (Schneider et al., 2006), ssmalloc (Liu and Chen, 2012), sfmalloc (Seo et al., 2011), SuperMalloc (Kuszmaul, 2015), or scalloc (Aigner et al., 2015).

Most modern operating systems such as Linux support the use of alternative allocators without any changes to the code of the application by providing a preloading mechanism for shared libraries (Kerrisk, 2010, Ch. 42.5), typically by setting the environment variable LD_PRELOAD to the shared library file of the allocator. Alternatively, one can link the alternative allocator statically to the application; this may require additional linker flags to allow overriding of functions, e.g., -Wl,--allow-multiple-definition on IBM BlueGene/Q systems.

### 2.5. Benchmark protocol

We obtain the quantitative results reported here by building and simulating the exemplary network model (Section 2.2.2) on two different computing systems (Section 2.2.1) with corresponding network sizes as specified in Table 1. In the following, one execution of the network model is called a benchmark run. We measure the execution time and the memory consumption at different stages of the benchmark run and compute statistics across multiple runs of the same configuration. If not stated otherwise, all data points are means of five benchmark runs and errors are the standard error of the mean.

**Figure 3:**
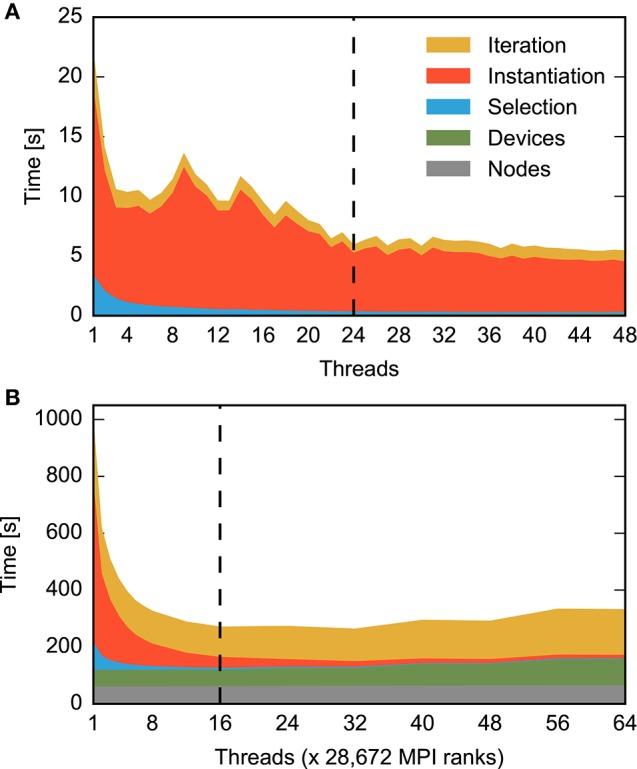
**Contributions to construction time for small and large neuronal networks for different numbers of threads**. The time required for different construction steps is indicated by color and stacked: time for creation of network nodes (gray) and connecting devices to neurons (green) are shown in total, while the time for connecting neurons to each other using the fixed-indegree algorithm is further divided into the random selection of sources (blue), the instantiation of individual connections (red), and iteration over nodes (yellow). Listing 1 shows correspondingly colored code for the latter three components. A network of fixed size is distributed across increasing numbers of OpenMP threads (strong scaling). The dashed vertical line indicates the number of physical cores available on the compute node. Network construction is performed with instrumented code incurring runtime overhead compared to Figures 1, **5**, **7**, see Section 2.5. **(A)** Construction of the small benchmark. Times required for creation of network nodes and for connections between devices and neurons are not visible. Same network model and machine as in Figure 1. **(B)** Construction of a balanced random network model representing 200 million neurons and 2.25 trillion synapses (large benchmark, Table 1, detailed model specification in Section 2.2.2) on an IBM BlueGene/Q supercomputer (Table 1, detailed system specification in Section 2.2.1). The benchmark emulates distributed computing on a constant number of 28, 672 compute nodes coupled by MPI while the number of OpenMP threads on each node is increased using the dryrun feature of NEST (Kunkel et al., 2014).

The execution time of a benchmark run is measured at two levels of detail. In the benchmark script, we use the NEST commands tic and toc to obtain the time spent in different sections of the code: creation of network nodes, creation of connections, and propagation of the dynamical state for the specified span of biological time. The commands are implemented using the POSIX function times() (Kerrisk, 2010), providing approximately millisecond precision. To obtain more fine-grained execution times of individual parts of the network construction process at the C++-level, we use the Stopwatch class provided as part of NEST. Stopwatch measures time using the POSIX gettimeofday() function (Kerrisk, 2010), which approximately provides microsecond precision. Since gettimeofday() incurs considerable run-time overhead, especially on IBM BlueGene/Q, we use code instrumented with Stopwatch only to dissect network construction times (Figures 3, **6**), while all other figures are based on data from un-instrumented code.

The memory consumption of a benchmark run is measured using the NEST command memory_thisjob. On a Linux operating system, such as the one investigated in the small benchmark (Table 1), this returns the current resident set size (VmRSS), i.e., the section of virtual memory that is mapped into the main memory (RAM, see Kerrisk, 2010). On the system under study in the large network benchmark (IBM BlueGene/Q, Table 1), the function returns the heap and stack size using the kernel function Kernel_GetMemorySize() and we report their sum as the memory consumption.

The small network model is executed on a single compute node. A benchmark run either systematically increases the number of threads or the number of MPI processes, from a single-threaded simulation to the maximal degree of parallelization supported by the hardware. The different allocators are evaluated by preloading them into the simulation engine as a shared library (see Section 2.4) overriding the standard functions malloc() and free() as described above.

We assume that the large network model is distributed over a large number of compute nodes, running one MPI process each. As each compute node constructs an equal share of the network and network creation is independent of MPI communication, a single compute node is sufficient for gathering the data on memory consumption and construction time. We therefore use the dryrun mode of NEST (Kunkel et al., 2014), which prepares the simulation engine for a distributed simulation but starts only a single process. For a constant number of MPI processes the benchmark run again systematically increases the number of threads on the compute node. Here the different allocators are tested by statically linking them to the simulation engine, overriding the standard functions malloc() and free().

## 3. Results

Figure 3 shows the runtime contributions of different sections of the network construction code for an increasing number of threads per compute node; due to additional instrumentation as described in Section 2.5, total times shown here are larger than the corresponding times shown in Figures 1, **5**, **7**. The small as well as the large network example exhibit appreciable scaling only up to a small number of threads. The patterns of the distribution of runtime contributions reveal, however, that the reasons are entirely different. Most of the time required to construct the small network is spent on instantiating individual connections (Figure 3A, red), while the other components become negligible. For the large network, in contrast, node creation (Figure 3B, gray), device connection (green) and the iteration component of the fixed-indegree algorithm (yellow) require constant or increasing time with growing number of threads.

The portion of the network handled by each of the 28, 672 compute nodes used for the large benchmark is comparable to the size of the small benchmark, with approximately 7, 000 neurons and 78 × 10^6^ synapses per node, compared to 25, 000 neurons and 62.5 × 10^6^ synapses in the small benchmark. Nevertheless, for the large benchmark network construction takes almost forty (39.8) times longer than for the small benchmark network (single thread, non-instrumented code).

These results suggest that the global network size imposes limits on the performance of the algorithm creating the part of the network local to the compute node. The hypothesis therefore is that the scaling limit observed for large networks (Figure 3B) overshadows the limit observed for small networks (Figure 3A). As both limits have a different origin, different patterns in the distribution of runtime contributions emerge.

In Section 3.1 we first investigate the nature of the bottleneck for small networks before we turn to the case of large networks in Section 3.2. Section 3.3 finally combines the optimizations resulting from the analysis at the two network sizes.

### 3.1. Limits imposed by memory allocation

In the small benchmark, the accumulated time of the connect() calls take up most of the time (Figure 3A). A detailed description of the connect algorithm is given in Section 2.3. In Section 2.3.2 we analyze the number of memory-related function calls involved in network construction. From Equations (3–5) and the parameters in Table 1, we obtain for *VP* = 48 virtual processes connector sizes *k*_EE_ ≈ 41.7 for excitatory-excitatory connections, *k*_EI_ ≈ 10.4 for excitatory-inhibitory connections and *k*_I_ ≈ 52.1 for all connections with inhibitory source neurons. Inserting into Equations (6) and (7) with *S*_E_ = ϵ*N* = 20, 000 excitatory and *S*_I_ = (1 − ϵ)*N* = 5, 000 inhibtory source neurons respectively and summing, we find that each virtual process must perform *n*alloc ≈ 325, 000 allocations and *n*free ≈ 280, 000 frees to construct the small benchmark network.

Thus, each virtual process performs over 600, 000 memory-related function calls in parallel with the other VPs. This points to memory allocation as a plausible candidate for the bottleneck in scaling.

**Figure 4:**
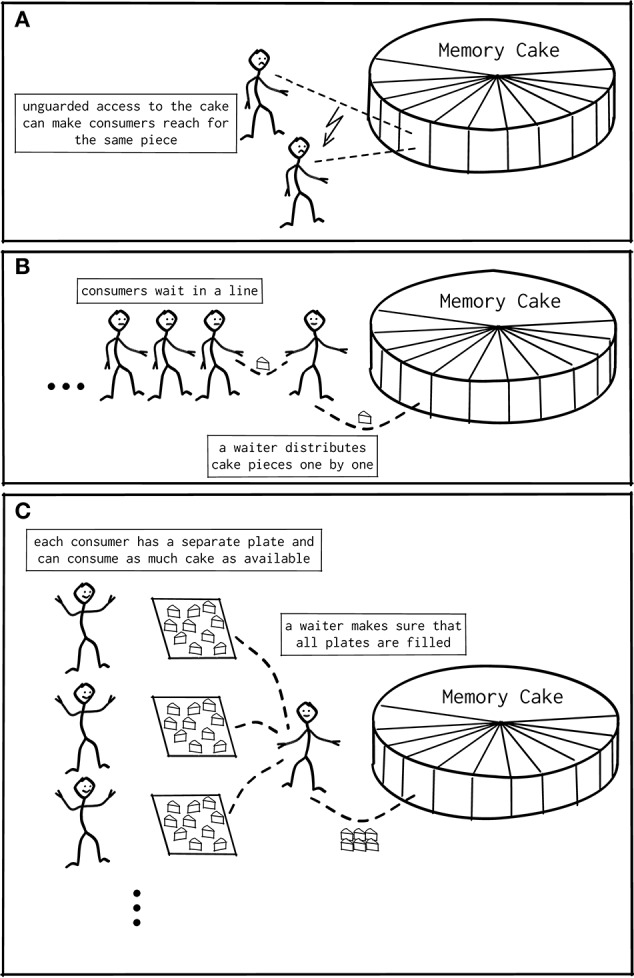
**Shared memory access patterns**. **(A)** Unguarded access to memory in multi-threaded programs can result in assigning the same memory region to different threads, causing unintentional overrides by the threads. **(B)** The trivial solution is to implement a guard or barrier managing all access to memory. This results in a bottleneck in case many threads access memory in rapid succession. **(C)** Modern memory managers implement thread-local regions, from which a thread can interact with memory: Interaction with the global memory becomes necessary only if the region is empty or overcrowded, and only this interaction needs to be guarded. This removes the bottleneck for most cases.

In case of serial or multi-process programs, or if there are only few memory related function calls, memory allocation performs well, as demonstrated by the blue curve in Figure 1B. In the multi-threaded case with many parallel allocations within a short time span, many threads idle, waiting for their turn to access the memory guarded by the standard implementation of malloc(). Figure 4B illustrates this situation. Indeed, explorative analysis with the HPCToolkit (Adhianto et al., 2010) indicates that calls to malloc() and free() require a large amount of time (data not shown). If this is true, improved memory allocation frameworks using ideas illustrated in Figure 4C should increase performance.

**Figure 5:**
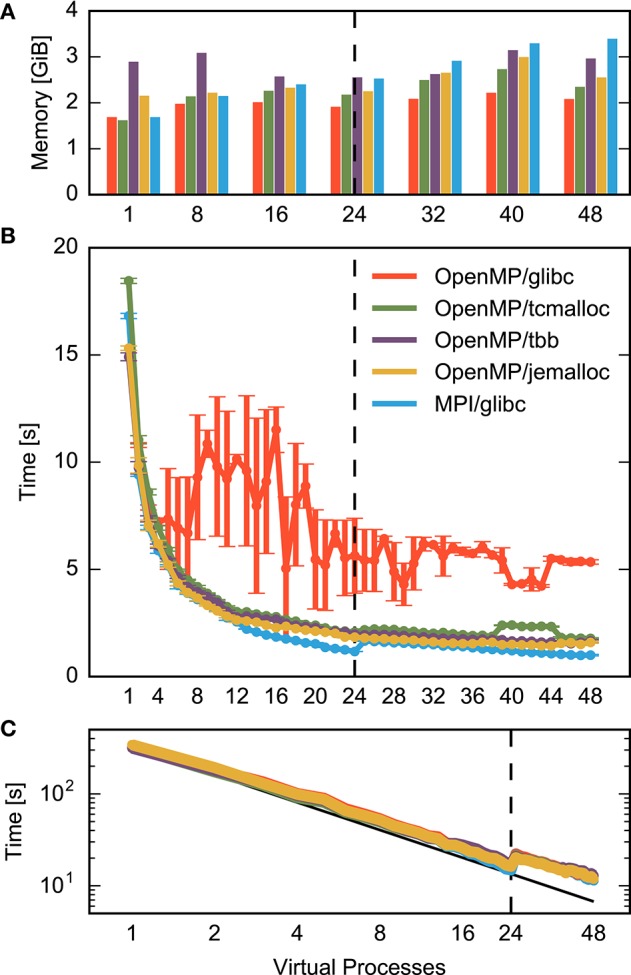
**Performance of a small neuronal network model using different memory allocators**. **(A)** Memory consumption after network construction, **(B)** runtime required for network construction, and **(C)** runtime for network simulation excluding network construction as a function of the degree of parallelization at constant network size (strong scaling). Data are for the same network model and machine as in Figure 1. The dashed vertical line in each figure indicates the number of cores available on the compute node. Results obtained with the standard allocator ptmalloc are shown in red (parallelization with OpenMP) and blue (parallelization with MPI) and show the same data as in Figure 1, while results for alternative allocation algorithms are shown in green (tcmalloc), violet (tbb), and yellow (jemalloc) respectively; see Section 2.4 for detailed specification of allocators. Data in **(C)** are shown in double-logarithmic representation, with the black line indicating ideal scaling.

Alternative allocators as described in Section 2.4 have primarily been developed for multi-threaded web services. Let us investigate how they perform under the conditions of a production code for the simulation of biological neuronal networks. Figure 5B summarizes the results for network construction. The standard allocator exhibits irregular behavior. After an initial reduction of runtime for two to four threads, runtime varies in a complicated manner until it stabilizes beyond 24 threads. The total speedup at 48 threads compared to the single-threaded application is just three (3.15). The allocators tcmalloc, tbb, and jemalloc all improve the performance to an almost proportional scaling as observed for parallelization by MPI. The latter scales well until all physical cores run one thread. The subsequent increase in runtime when the cores need to run two threads is only compensated toward the maximum number of threads supported by hyperthreading. In this limit, the well-scaling allocators reduce runtime by an order of magnitude (speed-up 9.6 for jemalloc using 48 threads), an improvement by a factor of three compared to the standard allocator. The variability across runs is vanishing. MPI still exhibits a superior 17-fold speed-up. Around 40 threads, some allocators show behavior deviating from the overall scaling trend, most likely due to details of the interaction between model structure, memory allocation and NUMA hardware.

The memory consumption (bars in Figure 5A) of the well-scaling allocators is larger than the consumption of the standard ptmalloc allocator, because the private memory regions require pre-allocation and thus a certain overhead per thread and may be less optimally exploited. With respect to MPI parallelization, however, multi-threading reduces memory consumption as the private regions require less memory than the overhead imposed by MPI (detailed in Figure 2).

The advanced allocators have techniques to support the creation of small objects (Section 2.4). These improvements are not relevant for the small benchmark scenario as all connections are stored in vectors, see parameter *N*^>1^_c_ in Table 1.

Independent of the choice of allocator or parallelization method, the scaling of runtime of the simulation of network dynamics time is close to optimal (Figure 5C). When the physical cores are exhausted, runtime increases as the simulation progresses according to the slowest thread. The neuronal network needs to be distributed over many more threads before a reduction in runtime is achieved compared to the value at the maximal number of physical cores. Overall, parallelization achieves a 27-fold speed-up in the simulation of network dynamics, bringing down simulation time from about 5.5 min to 12 s.

### 3.2. Limits imposed by loop order

Let us now turn to the situation for large neuronal networks. We already learned from Figure 3B that here runtime is not dominated by the command establishing the connection between two neurons. Other parts of the code creating the network consume the major fraction of runtime. Listing 3 shows the fixed in-degree algorithm introduced in Section 2.3. The colored ranges of code lines correspond to the distribution of runtime in Figure 3B. The outer loop iterates over all neurons in the network because all neurons receive synapses in this network. All threads are executing the loop in parallel, taking action only if a neuron is local. In a large neuronal network, however, fewer than one neuron in a million is local (*N*/*NVP* = 0.545, see Table 1 for parameters). Most iteration steps terminate immediately without doing any work. This extreme ratio suggests an alternative strategy that reverses the structure and loops over the much shorter list of local neurons for the price of a potentially more costly test. Listing 3 shows the alternative algorithm in its general form. The test needs to find out whether a given local neuron is in the list of target nodes. For our particular neuronal network this test is simple as the list of target nodes just contains all neurons in the network and can therefore be represented as a continuous range of integer values. The test is also efficient for more complex lists of target nodes if the list is represented by a suitable data structure such as a hash function or an ordered set.

**Listing 3:**
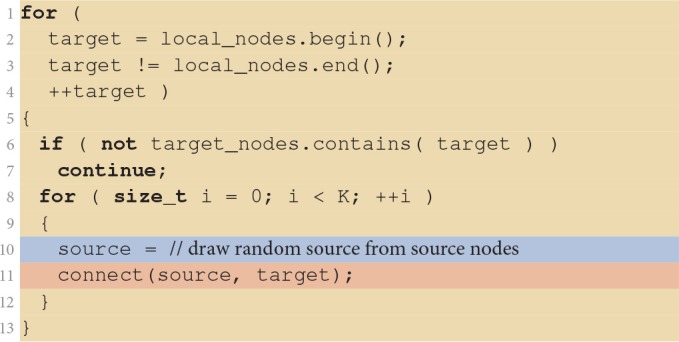
**Fixed in-degree algorithm with alternative loop order**. Compared to the original algorithm in Listing 1, the outer loop iterates over the local nodes (lines 1–4) and ignores all target neurons not in target_nodes (lines 6–7).

Figure 3B reveals that connecting devices to neurons and node creation also do not parallelize well. We address the former issue by reorganizing iteration code of the all-to-all connection algorithm, as well as other connection algorithms, along the same lines as the fixed-indegree algorithm. The flat node creation time observed for the large benchmark (Figure 3B, gray) is due to the fact that each virtual process needs to register some information about each of the 200 million neurons in the entire network to support source-target compatibility checks when creating connections (Kunkel et al., 2012). We improve the scaling of this process by registering non-local nodes en bloc instead of individually.

**Figure 6:**
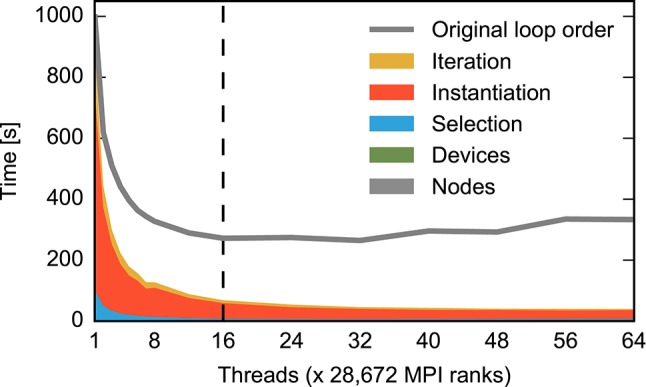
**Time required for the construction of the large neuronal network using the alternative loop order**. Color code and data ranges are the same as in Figure 3B showing the construction time prior to the reorganization of loops. The times required to create the network nodes and to connect devices and neurons are too small to be visible in the present graph. Listing 3 illustrates the algorithm. The gray curve shows the total construction time from Figure 3B for comparison. The dashed vertical line indicates the number of physical cores available on a compute node. The double-logarithmic representation of the same data in Figure 7 illustrates the scaling at large numbers of threads. Network construction is performed with instrumented code incurring runtime overhead compared to Figure 7, see Section 2.5.

**Figure 7:**
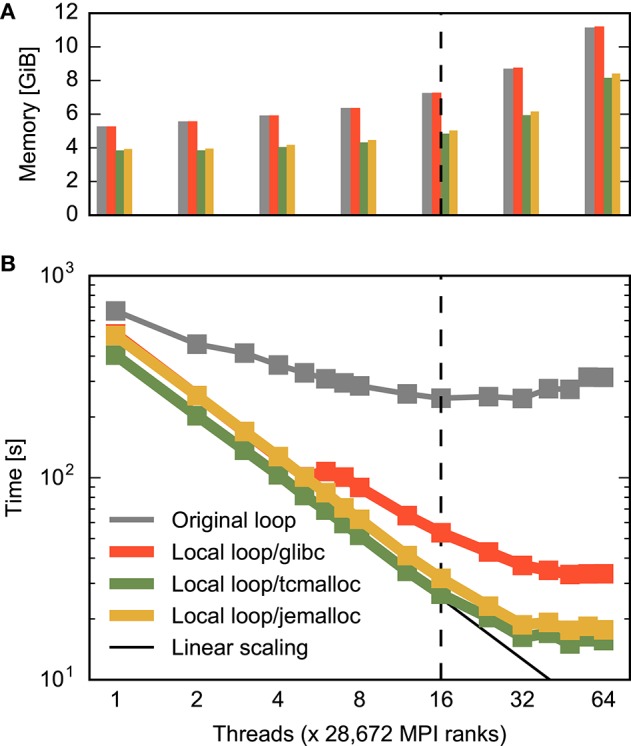
**Combined effect of improved memory allocation and loop order on construction time and memory consumption for a large neuronal network**. **(A)** Memory consumption at the end of and **(B)** time required for construction of the large network. The horizontal axis specifies the number of threads per MPI process as in Figure 3B. Gray bars/curve indicate the original implementation (same data as total time in Figure 3B), while red indicates results with loops carried out over local nodes (Listing 3, same data as total time in Figure 6). The green and yellow bars/curves combine this loop order with the memory allocators tcmalloc and jemalloc, respectively. The black curve in **(B)** indicates ideal scaling. The dashed vertical line indicates the number of physical cores available on a compute node.

Figure 6 shows the effect of the reorganization of loops based on measurements using instrumented code. The previously dominating parts of the code have vanished from the distribution of runtime. The overall effect is shown in Figure 7 for uninstrumented code (red vs. gray curve): When all 16 physical cores are in use, runtime reduces to a fifth compared to the original situation (from 248 *s* down to 53 *s*). At the maximum number of hardware threads runtime even improves by nearly an order of magnitude (from 314 *s* down to 33 *s*). In this case, the excessive iterations with increasing number of threads lead to an increasing runtime in the original algorithm while the runtime of the remaining components continues to decrease.

The resulting pattern of the distribution of runtime now resembles the one of the small neuronal network (Figure 3). The connect calls dominate and because of the reasons explained in Section 3.1 prevent a further reduction of runtime with increasing number of threads. Loop order does not affect the network construction time for the small network (data not shown).

### 3.3. Combined optimization of memory allocation and loop order

Figure 7 shows the combined effect of the improved multi-threaded memory allocation and the reorganization of loops discussed in the previous two sections on the construction of the large neuronal network. With the help of the advanced memory allocators, runtime decreases linearly until the 16 physical cores are exhausted, then decreases slightly slower until each core runs two threads and improves minimally beyond this point. Ultimately, runtime is about half of what is achievable with a reorganization of loops only (from 33 *s* down to 16 *s*). The use of multi-threading on the compute nodes of the supercomputer leads to a speed-up of 26 compared to the usage of a single thread per compute node. This is the difference between 7 min and just 15 s spent on network construction.

For the large network the advanced allocators also improve memory usage. Generally, memory consumption more than doubles due to administrative overhead when scaling from one to the maximal number of threads. Advanced allocators reduce the administrative overhead, so that memory consumption at the maximum number of hardware threads is 27% or 3 *GiB* lower using tcmalloc than using the standard allocator. Consequently, with the same amount of memory available, the number of neurons on a compute node can be increased by more than a third (1.375): four instead of three neurons can be represented. This is achieved by a better handling of large numbers of small objects by the advanced allocators (see Section 2.4).

## 4. Discussion

Since Dennard scaling enabling the steady increase in processor clock speed came to an end a decade ago (Chang et al., 2010), progress in compute power comes from multi-core architectures. Computational neuroscience has taken up the challenge to develop simulation code for spiking neuronal networks coping with the increasing parallelism of the hardware.

Today, the dynamics of even rather small networks of the size of a cortical microcolumn ($O\left(10^{5}\right)$ neurons) can be simulated with highly parallel code reducing the time required for the simulation of one second of biological time from several minutes to a few seconds. For brain-scale networks, petascale parallelization is essential to aggregate the main memory required to represent trillions of synapses. The success of the community's efforts in developing technology for the parallel execution of the dynamics rests on the natural microscopic parallelism of the dynamics of neuronal networks: at a common level of description in computational neuroscience, nerve cells are independent dynamical systems interacting with each other only by delayed point-like events.

However, before a particular neuronal network can be simulated it needs to be instantiated in the memory of the computer system. If this phase of the simulation does not parallelize to the same degree as the phase concerned with the dynamics of the network, network construction ultimately limits the scaling of the application. Just at the time when Dennard scaling ended, researchers pointed out the importance of parallel network construction (Morrison et al., 2005). The Message Passing Interface (MPI) provided a mechanism to distribute a neuronal network simulation over the compute nodes of high-performance clusters. For the small number of processors per compute node and the small network sizes considered, the resulting code indeed showed excellent scaling of network construction on clusters and shared memory machines. Consequently this software architecture became the backbone of spiking neuronal networks simulators.

In the present study we uncover fundamental limitations in the parallelization of network construction on cluster nodes with many compute cores and for networks orders of magnitude larger than previously considered.

For small networks (we remind the reader that small in this context means 25, 000 neurons) simulated on a modern compute node, we find excellent strong scaling up to the limits of the multi-core architecture when using MPI for parallelization, although at the price of significant memory overhead (almost 50%). Using OpenMP threads for parallelization instead avoids the memory overhead, scales perfectly in the simulation of network dynamics, but does not scale beyond four parallel processes in network construction. This is disappointing as CPUs already have dozens of compute cores each equipped with hardware supporting multiple threads while fast memory remains limited. It is therefore essential to find technologies exploiting multi-core architectures without the growing memory overhead which parallelization by MPI entails.

Our study traces the lack of scaling of OpenMP in the construction phase of small networks to memory allocation: Constructing the adjacency tables representing network connectivity requires a large number of small object allocations and deallocations. When more than four threads perform such allocations and deallocations simultaneously, the ptmalloc memory allocator used by default in current Unix-based systems, significantly slows parallel construction as all threads need to synchronize every time a single thread obtains or returns memory. Using modern allocators optimized for multi-threaded memory operations, such as tcmalloc, jemalloc, and tbb, practically eliminates the locking between threads and restores scaling.

An alternative approach to reducing thread contention due to memory allocation is to reduce the number of allocations and frees by creating each connector object as a dynamically-sized container with sufficient memory capacity as soon as the first synapse is registered for any source neuron. Tests with a pre-allocation of 64 elements showed better performance for intermediate thread numbers but no advantages for large numbers of threads and signficantly worse performance than when using modern allocators without pre-allocation (data not shown).

The absolute performance of thread-based parallelization is still 60% worse than MPI-based parallelization for more than twelve processes, see Figure 3B for 24 and 48 virtual processes. The origin of this effect is not yet fully understood, but may be related to the non-uniform memory access (NUMA) architecture of modern computers (Hager and Wellein, 2011, Ch. 4): Each processor has local main memory but can also access the local memory of other processors, albeit with higher latency. For an MPI-program, the operating system usually attempts to hold all data for each process in the memory local to the CPU running the process. When a multi-threaded program runs on several CPUs, though, memory may be allocated by different threads in different parts of the main memory, requiring threads to access data in non-local memory, thus incurring delays.

For large, brain-scale networks simulated on supercomputers, thread-based parallelization is vital: these computers typically provide significantly less memory per compute core than HPC clusters and are thus de facto limited to a single MPI process per compute node. We find no or negative scaling beyond 16 threads per compute node. Here, however, the effect found for small networks is overshadowed by an entirely different problem. The connection algorithm spends most of its time iterating over potential target neurons it is ultimately not responsible for, thereby effectively serializing the processing. Reorganization of the loop order for the price of a slightly more complex test removes the serialization and excellent scaling is achieved up to the full number of threads supported by the hardware. Combining the new algorithm with allocators designed for multi-threading further improves the performance.

Overall, we have reduced network construction times for microcolumn-sized networks on 48 threads by a factor 3.4, from about 5.3 times to just 1.6 times slower than MPI, while reducing memory consumption by about 25% compared to MPI. At this level of parallelization, network construction now contributes only 1.6 s to the total runtime while it takes 11 s to simulate 1 s of biological activity. Further improvements will thus have less impact. Reducing the network construction times also leads to, albeit minimally, improved times for network simulation, as indicated by Figure 5C.

The improvements for the large (brain-scale) benchmark are more significant: The original code requires about 247 s to construct the brain-scale network in the best case (32 threads). After optimization, the code scales much further, constructing the network in just 16 s using 64 threads, an improvement by more than a factor of 17. This difference matters: It reduces the time required to construct a brain-scale network on the entire supercomputer by nearly 4 min, or about 30, 000 core hours, almost 1% of a typical 5 million core hour allocation for a supercomputing project. Brain-scale simulations on supercomputers are not feasible without the improvements in network construction presented here.

The quantitative data shown here are obtained using a concrete implementation of network construction and simulation code in the NEST simulator. The conclusions obtained are however generic. The instantiation of model neurons takes up negligible time and models are only distinguished by the number of state variables. Therefore, our results also hold for more complex neuron models than the integrate-and-fire model used throughout the study. The creation of synapses contributes considerably to the time required for network creation. With respect to network creation, synapse models supporting long or short-term plasticity differ from static synapses only in the number of state variables per synapse. Consequently, there is no effect on the scaling of network creation. More complex network structures typically require a larger number of calls of high-level connectivity routines issued from the serial simulation script. This does not reduce the improvement of allocation but an additional serial component reduces the total gain in performance.

The advantage of MPI parallelization over multi-threading observed previously is not due to a typical runtime vs. memory consumption dilemma. Multi-threaded code achieves excellent scaling when used with modern memory allocators at considerably lower memory consumption than MPI. In brain-scale network simulations, the number of neurons represented on a single compute node is orders of magnitude smaller than the total number of neurons in the entire network. Connection-generating algorithms need to be designed such that loops extend over local elements only, independent of whether parallelization is realized using MPI or multithreading.

## Author contributions

The authors jointly worked on all aspects of the study and the preparation of the manuscript under the supervision of HEP and MD. The code analysis, coding, and data gathering was mainly done by TI guided by JME and HEP.

### Conflict of interest statement

The authors declare that the research was conducted in the absence of any commercial or financial relationships that could be construed as a potential conflict of interest.
